# Draft Genome Sequence of a Chryseobacterium indologenes Strain Isolated from a Blood Culture of a Hospitalized Child in Antananarivo, Madagascar

**DOI:** 10.1128/MRA.00752-19

**Published:** 2019-08-29

**Authors:** Mamitina Alain Noah Rabenandrasana, Lala Fanomezantsoa Rafetrarivony, Lalainasoa Odile Rivoarilala, Vincent Enouf, Annick Lalaina Robinson, Ando Rakotozanany, Jessica Vanhomwegen, Valérie Caro, Cassandre Von Platen, Jean-Claude Manuguerra, Jean-Marc Collard

**Affiliations:** a Experimental Bacteriology Unit, Institut Pasteur de Madagascar, Antananarivo, Madagascar; b Laboratory for Urgent Response to Biological Threats, Institut Pasteur, Paris, France; c Center for Translational Science, Institut Pasteur, Paris, France; d Centre Hospitalier Universitaire Mère-Enfant Tsaralalàna (CHUMET), Antananarivo, Madagascar; e Pasteur International Bioresources Network (PIBnet), Plateforme de Microbiologie Mutualisée (P2M), Institut Pasteur, Paris, France; Loyola University Chicago

## Abstract

We report here the draft genome sequence of a Chryseobacterium indologenes strain, isolated from a blood culture of a 2.2-year-old child admitted to the hospital for vomiting and coughing. The genome was composed of 5,063,674 bp and had 37.04% GC content. We detected 4,796 genes with predicted protein-coding functions, including those associated with antibiotic resistance.

## ANNOUNCEMENT

Bacteria of the Chryseobacterium genus are nonmotile, chemoorganotrophic, and glucose-nonfermentative Gram-negative rod-shaped bacteria. The most pathogenic species of the genus, Chryseobacterium meningosepticum, which causes numerous infections, was reclassified to the genus Elizabethkingia (1). Chryseobacterium indologenes, although ubiquitous in nature and found mainly in soil and water, is an uncommon human pathogen. However, in rare cases, it can cause serious infections, particularly among immunocompromised hospitalized patients with severe underlying diseases and/or with indwelling catheters (2). These include keratitis, bacteremia (2), pneumonia, cellulitis, and artificial shunt infection (3–6).

C. indologenes was isolated from a positive blood culture (time to positivity, 23 h) collected by venipuncture from a 2.2-year-old child hospitalized at the pediatric hospital of Tsaralalàna, Antananarivo, Madagascar. This child was admitted 1 day before blood sampling with fever, coughing, and vomiting. The study was approved by the Ministry of Health and the National Ethics Committee of Madagascar (number 140-MSANP/CE). Informed written consent was obtained from at least one child’s parent before sampling.

The strain forms on solid blood agar in typical yellow colonies due to a flexirubin-type pigment. After 24 h, the colonies were identified by matrix-assisted laser desorption ionization–time of flight mass spectrometry (MALDI-TOF MS) as *C*. *indologenes*, with a score of >2.0. The antimicrobial susceptibility testing was performed according to the standard disc methods described in the CA-SFM guidelines (7), and susceptibility to chloramphenicol and tetracycline was also tested. The isolate was resistant to ticarcillin, piperacillin, ticarcillin plus clavulanate, tazobactam, ceftazidime, aztreonam, imipenem, gentamicin, tobramycin, amikacin, and ciprofloxacin, displayed an intermediate resistance to chloramphenicol, and remained susceptible to cefepime and tetracycline.

DNA extraction was done from an overnight culture using a DNA blood and tissue kit (Qiagen, France). A DNA sequencing library was prepared using a Nextera XT DNA sample prep kit (Illumina, Inc., San Diego, CA), according to the manufacturer’s instructions. Genome sequencing was performed using MiSeq Illumina technology (2 × 300 bp), generating 3,772,882 paired-end reads. FqCleaner version 3.0 was used to eliminate adapter sequences (8, 9), reduce redundant or overrepresented reads (10), correct sequencing errors (11), merge overlapping paired reads, and discard reads with a Phred score (measure of the quality of identification of nucleobases generated by automated DNA sequencing) of <20. Illumina read *de novo* assembly was performed using SPAdes version 3.10.0 (12, 13), with default parameters. Acquired resistance genes were detected using ResFinder version 3.0 (14). The genome was annotated using the PATRIC Web server version 3.5.39 (15).

In total, 48 assembled contigs with an *N*_50_ value of 193,241 bp and an average coverage of 36.1×, which had 5,063,674 bp and 37.04% GC content, were annotated using the PATRIC Web server annotation pipeline (15–19). The genome harbors 64 tRNA genes, 3 rRNA genes, and 4,796 protein-coding sequences. The antimicrobial resistance (AMR) genes detected in this genome and the corresponding AMR mechanisms are provided in Table 1.

**TABLE 1:** Antimicrobial resistance genes found in a *C. indologenes* strain isolated in Antananarivo, Madagascar

Resistance gene	Function	Classification
*tet*(X)	Tetracycline-inactivating enzyme	Antibiotic inactivation enzyme
*bla*_IND-2a_	Subclass B1 β-lactamase	Antibiotic inactivation enzyme
*katG*	Catalase peroxidase	Antibiotic inactivation enzyme
*ant(6)-I*	Aminoglycoside 6-nucleotidyltransferase	Antibiotic inactivation enzyme
*bla*_CIA_	Class A extended-spectrum β-lactamase	Antibiotic inactivation enzyme
*catB*	Chloramphenicol *O*-acetyltransferase	Antibiotic inactivation enzyme
*oxyR*	Hydrogen peroxide-inducible gene activator	Regulator modulating expression of antibiotic resistance genes

The extended-spectrum β-lactamase gene *bla*_CIA_ was responsible for the resistance to ticarcillin, piperacillin, ticarcillin plus clavulanate, tazobactam, and ceftazidime but not to cefepime. A metallo-β-lactamase gene, *bla*_IND-2a_, was responsible for the resistance to imipenem. Our phenotypic results showed that the *catB* gene did not confer complete resistance to chloramphenicol and that the *tet*(X) gene was nonfunctional, like that originally found in Bacteroides spp. (20–23). The Tet(X) protein showed 62% identity with Tet(X) from the strain AF25-18 (GenBank accession number RJV36860) and 62% with Tet(X) from Chryseobacterium oncorhynchi (GenBank accession number PWN64762). Studies on hosted bacteriophages, secretion systems, mobility, and ability to form biofilms would allow us to understand the pathogenic potential of our *C. indologenes* isolate and its role in bacteremia.

### Data availability.

This whole-genome shotgun project has been deposited at DDBJ/ENA/GenBank under the accession number VCBR00000000. The version described in this paper is version VCBR01000000. Raw sequence data for this strain were deposited under SRA accession number PRJNA551338.
